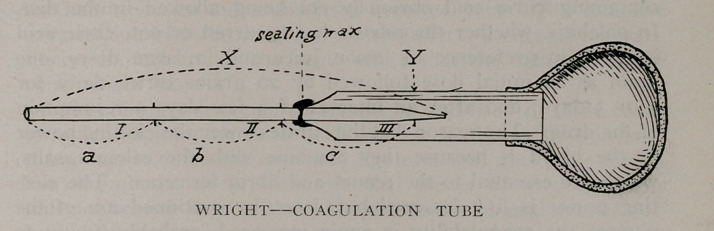# Varicose Veins

**Published:** 1908-06

**Authors:** James A. Macleod

**Affiliations:** Buffalo, N. Y. 327 Delaware Avenue


### BUFFALO MEDICAL JOURNAL.

Vol. Lxiii. JUNE, 1908. No. 11

### ORIGINAL COMMUNICATIONS.

### Varicose Veins.

By JAMES A. MACLEOD, M. D., Buffalo, N. Y.

THE condition of varicosity of veins, in whatever part of the body it may exist has, in recent years, gradually been assuming an ever increasing importance in the consideration of the public and of the surgeon. For this great interest two well grounded facts may be assigned. First, the insistence of the Civil Service Commission, the Army and the Navy that those presenting themselves before the medical examiners must be free from any form of varicosity. Second, the great increase in the number of cases in the last half century, due to the congregation and congestion in cities of large masses of people living a confined, non-hygienic life devoid of exercise and lacking proper nourishment. Great numbers of young men and women, scarcely matured, preferring the excitement of city life to that of the more healthy rural, are compelled to accept employment in the large departmental stores. The wage paid is barely sufficient, even with the exercise of judgment and the strictest economy, to supply all the requirements of a healthy body, but foresight is not as a rule displayed by such individuals. The exciting cause that brought them to the city sways them in wasting of their earnings. Long days spent entirely on their feet are followed by late hours passed in crowded theatres and dancing halls. Anemia naturally follows, the whole system is weakened and with it the veins, any slight exciting cause—a circular garter—is sufficient to cause the mischief.

The same applies, though to a lesser degree, to conductors on street cars and railroad trains, motormen, nurses and the like. Finally, the rapidly increasing prevalence of rickets,—resulting largely from the above conditions,—in London, New York and all great cities play an important part in the subsequent causation of varicosity. Deformities of the pelvis, sequelæ of the rickety condition of childhood, lead to difficult labors, to mis-

1. Read before the Gross Medical Club April 24, 1908.

placed uteri, to added pressure upon the great veins during pregnancy; the exciting cause being thus present, the condition of varicosity soon obtains.

Neurasthenia, in former years rarely seen, is very prevalent from the high tension at which the present day American lives. Slight and insignificant pains are magnified into great, and especially is this true where the genitals are concerned. The young man with a small varicocele, occasionally feeling uncomfortable, soon distorts it into something of grave import; even in some instances to such magnitude that the surgeon feels compelled to operate upon the veins for the cure of the boy's mental state.

By the term varicose vein is meant a permanent dilatation and lengthening, leading to more or less tortuosity of the vein, the walls are thickened, the tunica intima and the tunica media the most markedly so; the capsule and, in some cases. the surrounding tissues may also undergo like sclerosing; the valves atrophy and become functionless; in some instances the dilatation is not uniform but pouch-like in places; these pouches may be so marked along the course of the vein as to give rise to definite tumor-like bodies, designated venous cysts.

The condition of varicosity may be found not only among the superficial veins, but also among the deep ones. The common sites for varicosity are the superficial veins of the lower extremities, the veins of the pampiniform plexus of the spermatic cord and the lower hemorrhoidal veins of the rectum. Varicosity has two main factors in its causation:

(*a*) Increased intravenous pressure; this is, undoubtedly, the more important factor and is always due to back pressure.

(*b*) Weakness of the vein wall; this may be inherited or acquired, where it is acquired, as it usually is, it is always secondary to increased intravenous pressure.

The causes of increased intravenous pressure may be classified under two headings:

1. Causes arising in the lumen of the vein.
2. Causes arising external to the vein.

First, causes arising in the lumen of the vein. The actual weight of a column of blood, where the valves are weak or absent, may cause varicosity of the distal veins, and this is seen in people whose occupation necessitates their standing on their feet the greater part of the day. Where a vein is dilated the valves, as pointed out above, become useless and the superincumbent weight of blood tends to further increase the dilatation by the force of gravity. Thrombosis of a main vein may, and usually does, cause varicosity of the veins distal to the thrombosis; if the inferior vena cava becomes thrombosed, the mammary and the epigastric veins become varicose and stand out prominently on the surface of the anterior abdominal wall. General venous obstruction, as in the case of chronic cardiac and pulmonary disease, and portal obstruction, as in the case of hepatic congestion or cirrhosis, may act as factors in the production of varicosity.

Second, causes arising external to the vein. These causes act by direct pressure on the vein, and may be enumerated as follows:

(*a*) Tight garters.

(*b*) An illfitting truss.

(*c*) Pelvic tumors of either pathological or physiological character.

(*d*) Tumors deep in the abdomen.

(*e*) Enlarged glands, malignant or otherwise.

(*f*) Excessive muscular development, causing pressure on the deep veins, as is seen in the lower extremities.

(*g*) Chronic constipation.

(*h*) Scar tissue.

This condition of varicosity may be divided into three classes: (1) hemorrhoids; (2) varicocele; (3) varicose veins.

*1.. Hemorrhoids.*—I will not give more than passing mention to the discussion of hemorrhoids in this paper; let it suffice to say that by the term hemorrhoids is meant a varicosity of the lower hemorrhoidal veins of the rectum.

*2. Varicocele.*—By the term varicocele is meant a varicosity of the veins of the pampiniform plexus of the spermatic vein. It is more commonly present on the left side, and this may be explained by the fact that the left spermatic vein opens into the left renal vein without a valve at its orifice, whereas the right spermatic vein opens directly into the inferior vena cava and is valved at its orifice. Pressure on the left spermatic vein by the sigmoid flexure, in constipation, may also tend to make the varicosity more commonly present on the left side. Pressure on the renal or spermatic veins by a tumor of the kidney, or by glands arising from such, may cause a varix; varicocele appearing late in life should give rise to a strong suspicion of malignant disease of the kidney on the same side as the varicocele.

A varicocele is characterised by a distinct tumor-like body in the scrotum, which disappears upon the patient assuming the recumbent position and which, again, becomes evident upon the patient resuming the erect position; there is a distinct impulse on coughing. It is exceedingly common and may, or may not, give rise to symptoms; if present, the symptoms consist of a sense of fulness, of weight, of heat and, even, a sense of pain referable to the testicle.

The diagnosis is usually simple, as the sensation imparted to the examining finger is quite characteristic, and is likened to a bag full of worms. The only condition likely to be mistaken for a varicocele is an omental inguinal hernia extending down into the scrotum; each gives rise to a distinct impulse on coughing; each disappears upon the patient assuming the recumbent position, but if the finger be placed firmly over the external abdominal ring and the patient be made to stand up, the tumor in the case of the hernia does not reappear; whereas in the case of the varicocele it again becomes evident, the veins filling from below.

The treatment may be palliative or operative. The palliative consists in keeping the bowels active, and the wearing of a suspensory bandage; such measures should be given a fair trial before thinking of operative procedures, except where the patient is barred from entering some occupation by the presence of the varicocele. If operative treatment be decided upon, the only method to be considered is the open one, and in it the incision is preferably made over the external abdominal ring.

*3. Varicose Veins.*—By the term varicose veins is meant a varicosity of the veins, otherwise than of the hemorrhoidal and spermatic ones, and is more especially applied to the permanent dilatation and lengthening of the superficial veins. Varicose veins are most commonly found among the superficial veins of the lower extremities. Symptoms may or may not be present; when present, they consist of a sense of weight, of heat, of discomfort and even, in some cases, a sense of pain.

The diagnosis is usually simple, as the veins stand out prominently on the surface of the limb; they usually, subside upon the patient assuming the recumbent position. The most likely condition to be confused is a femoral hernia presenting at the saphenous opening, and then only in the case of a venous cyst at that situation. I remember one patient being sent into the hospital for operation for femoral hernia, which, on examination, proved to be a venous cyst; we have in each a distinct impulse on coughing; each of them may disappear upon the patient assuming the recumbent position, but if the finger be placed firmly over the femoral canal and the patient be made to stand up, the tumor, in the case of the hernia, does not become evident; whereas, in the case of the venous cyst, it reappears, the cyst filling from below.

*Complications.*—(*a*) Pigmentation of the skin, where the veins are small and numerous, is a very common occurrence, especially in those cases, which have been complicated by eczematous states.

(*b*) Phlebitis is a very common occurrence, and may end in resolution or thrombosis. In some cases we have recurrent attacks of inflammation, and in such the vein walls become very markedly thickened, so much so that the vein can be readily mapped out, even with the patient in the recumbent position.

(*c*) Thrombosis most commonly occurs as a sequela of phlebitis, but it may also ocur in conditions, in which we have a stasis of the venous current, for example, in the venous pouches or ampullæ of the valves. Organisation of the clot may take place, and go a long way toward affecting a cure of the varix. In some cases we may even have calcification of the clot; I have seen a number of small calcareous phleboliths in operating for varicose veins. The clot may become detached and swept into the general circulation, where it may lead to serious mischief through pulmonary or cardiac embolism. The clot, especially where the thrombosis has been caused by a phlebitis, may break down and suppurate; the suppuration may be very extensive, involving not only the vein itself, but also the surrounding superficial tissues; it may spread along the vein, being preceded by thrombosis, into the deep veins, where it may lead to wide-spread mischief. Pieces of septic clot may become detached in the venous current causing in the case of the portal circulation multiple hepatic abscesses, in that of the general circulation a general pyemic condition with its many embolic abscesses.

(*d*) Eczema is very prone to occur as a complication, on account of the congested state of the skin.

(*e*) Ulcers of the skin are also very prone to occur, especially in cases already complicated by eczema.

(*f*) Hemorrhage may occur from injury, or from ulceration; when present, it is usually very copious, the bleeding taking place from both ends of the severed or perforated vein, the valves above not acting.

The treatment hinges largely on the causative factor or factors of the varicosity, and on the complications, if any be present. We may divide the question of treatment into eight classes, namely:

1. Cases in which there is no gross obstruction.
2. Cases in which there is a gross obstruction.
3. Cases which are complicated by the presence of venous pouches.
4. Cases which are complicated by the presence of eczema.
5. Cases which are complicated by the presence of ulcers.
6. Cases which are complicated by the presence of phlebitis.
7. Cases which are complicated by the presence of thrombosis.

8. Cases which are complicated by the occurrence of hemorrhage.

*Group 1.—Cases in which there is no gross obstruction.*—The varix in these may be the result of a congenital weakness of the vein walls, but such a defect is, usually, only one of the factors in the production of the condition; the common causes, to which the above mentioned weakness may be concurrent, are as follows: prolonged standing, as is seen in the case of street car conductors; compression of the deep veins by excessive muscular development, as is seen in the case of athletes, and the wearing of tight garters or an ill-fitting truss.

Symptoms may, or may not be present; if present, palliative measures should be given a fair trial; the causative factor or factors should be removed, where feasible; if a truss be worn, a properly fitting one should be procured, an elastic bandage worn, and a general tonic treatment prescribed. Failing these measures, and even where these give relief it may be necessary to remove the veins, to insure the patient's admission to certain occupations, for example, the army, the navy, and the civil services.

The operations for the relief of varicose veins are numerous, but the most popular are as follows: (1) Excision of the entire involved vein. (2) Excision of a small portion of the varicosed vein, close to its junction with the main vein, or even a simple ligation of it at the same position, for example, at the saphenous opening, when the internal saphenous vein is involved. (3) Excision of small portions of the vein at junction with its tributaries. (4) A circular incision of the limb down to the deep fascia, tying off all veins encountered.

I have tried all of the above methods of operation, and my best results have been obtained where I have employed the third one—namely, excision of small portions of the vein at junctions with its tributaries. My mode of procedure is as follows: the patient having been anesthetised, and the limb purified, a tourniquet is applied well above the site of the varx; the veins, which become engorged and stand out prominently on the surface of the limb, are marked at desirable points on the skin, picking out the main junctions, by light touches of a sharp scapel. The tourniquet is now removed, and the limb held up to drain the engorged veins of blood. An incision is made down to the vein at each position marked, and about one inch of it excised, tying off at the same time a small portion of the tributary. In a number of cases I have seen other veins become prominent a few months after the operation; these have been treated in a similar manner by a secondary operation

*Group 2—Cases in which there is a gross obstruction.*—The treatment here depends entirely on what the obstruction may be. It may be taken, however, as a cardinal rule that no operation upon the veins should be undertaken whilst the obstruction persists. If it is feasible to remove the obstruction, and if after its removal the varix still persists then operation toward its relief is in order. Varicosity complicating pregnancy subsides, as a rule, after the pregnancy is terminated.

*Group 3—Cases which are complicated by the presence of venous pouches.*—These pouches, the walls of which are thin, stand out prominently and are thus very liable to injury. The hemorrhage from a ruptured venous cyst is always very copious, in fact so much so as to endanger the life of the patient. The presence of a venous cyst or pouch is, therefore, always an indication for operation. The cyst should be excised, and the rest of the mischief treated in a manner similar to that described above.

*Group 4—Cases which are complicated by the presence of eczema.*—In the treatment of these I have found elevation of the limb, and the application of Unna's dressing to be the most efficacious.

*Group 5—Cases which are complicated by the presence of ulcers.*—These ulcers are usually of an indolent character; the base is more or less adherent to the underlying tissues; the edges are hard, sharp cut, and elevated above the rest of the surface; the surface is usually smooth and glistening, and of a dirty yellow appearance, with perhaps a few badly-formed granulations. These ulcers may be simply indolent, the discharge being a thin yellowish serum, or they may be very foul, the discharge being profuse and purulent. The treatment varies with the age and condition of the ulcer. In the early stages all that is necessary is rest and elevation of the limb, and protection of the ulcer from irritation. In the later stages the first object is to obtain as near an aseptic condition of the ulcer as possible, and I begin treatment, whether the condition is apparently clean or evidently unclean, by a course of antiseptic fomentations until I feel that the ulcer is in a fairly aseptic state. I then apply Unna's dressing, which is made up as follows: gelatine, 5 parts; oxide of zinc, 5 parts; boric acid, 1 part; glycerine, 1 part; water, 6 parts; to this is added a little ichthyol or sulphur. The limb is first washed with soap and water and purified with carbolic lotion (1 in 20). It is then wrapped round, from toes to knee, with a single layer of aseptic gauze, and the paste, heated so as to make it liquid, is applied over the gauze. Another layer of gauze is applied over the paste and the whole allowed to dry. This dressing is changed from time to time, varying from a couple of days to one week. Many cases rapidly heal under the above regime. Failing cure in a few weeks I have tried, with very gratifying results, methods along the line of treatment used by Bier. In this treatment we make use of a congestion, brought about by the wearing of a tight constricting band above the site of the ulceration. The patient is directed to use a Martin's bandage, applying it firmly and evenly just below the knee; he is directed to keep the limb elevated and wear the bandage for twenty minutes four times a day. The ulcer, in a large number of cases, rapidly fills up with strong healthy granulations and the epithelium proliferates over these.

Where the ulcers have become septic and do not readily clear up under ordinary surgical dressings great benefit is frequently observed from the use of bacterial vaccines. It is wise to make cultures and isolate the infecting organism; if it is a staphylococcus a stock vaccine of unquestionable value should be employed, but if it is a streptococcus a specific vaccine must be used. In cases so situated that cultures cannot be made it is justifiable to use a stock vaccine under the presumption that such cases are usually due to the staphylococcus but, in my opinion, if there is no marked improvement after the third inoculation, the treatment should be discontinued pending the isolation of the infecting organism. Where stock vaccines are employed, 100 millions of the staphylococcus aureous and 20 millions of the staphylococcus albus are injected twice a week. It is also well during the administration of the vaccines to increase the flow of serum to the part by reducing the clotting power of the blood, by means of therapeutic measures to be described later in this paper. The improvement under this regime is rapid up to a certain point, that is, up to the time of the cure of the septic part of the mischief. Thereafter we must rely on Unna's or Bier's method of treatment as depicted above, or on surgery.

Some cases are exceedingly obstinate, due to the indurated condition of the base, which is adherent to the underlying tissues, and even in some instances to the periosteum; it may be necessary in these to thoroughly curet the base and edges. I have, on several occasions, excised the whole ulcer; granulations usually spring up abundantly after the operation; it may be wise in some instances, where the ulceration is widespread, to skingraft at an early date. Some surgeons advise operating on the veins for the relief of the ulceration. If the operation is performed it should be deferred until the ulceration is healed up; even then it is questionable, in my opinion, whether or not it is a proper procedure. I have seen a number of cases in which the ulceration recurred after the removal of the veins, and in each it proved to be most intractable.

*Group 6—Cases which are complicated by the presence of phlebitis.*—In all cases of phlebitis the clotting power of the blood is high, that is, the blood clots in less than normal time. It is certainly beneficial, taking into consideration the further complication of thrombosis with all of its dangerous possibilities, to lower that power by adhering closely to the therapeutic measures presently to be described. The clotting power of the blood may be quickly ascertained by the laboratory technic employed by Ross, of Toronto. The apparatus—coagulation tube of Wright—and the method of investigation may be briefly described as follows: the coagulation tube of Wright comprises

WRIGHT—COAGULATION TUBE

x a fine capillary tube of such a caliber that 5 centimeters of its length just contains 5 cubic millimeters of blood. x is divided into three parts—a, b and c—by points I and II; part b is 5 centimeters in length and contains 5 cubic millimeters.

The contents of part a is equal to that of part c; y is a glass holder fixed by sealing wax on to the tube x; z is a rubber bulb.

The upper end of the tube x is made with such a fine caliber that whilst air can pass through mercury cannot. The technic employed is simply but rapidly performed. The rubber teat is compressed between the thumb and forefinger; mercury is drawn up to point I; the blood from a punctured finger is drawn up until the mercury reaches point II; the end of the tube is then removed from the blood and the pressure released from the teat; the mercury passes up the tube until it is stopped by the fineness of the caliber. It is obvious that we now have just 5 millimeters of blood occupying a space 5 centimeters in length in the tube. The tube now containing the mercury, blood, and air is immersed in a water bath with a temperature of 38 degrees centigrade. The normal clotting time of the blood in a tube so made and under the above conditions is from one and a half to two minutes. The tube, having been in the bath for one and a half minutes, is withdrawn and its contents rapidly expressed upon white blotting paper, and the condition of the blood observed; if fibrin is just commencing to form then one and a half minutes is the clotting time, if there is no sign of fibrin formation a fresh tube is prepared and placed in the bath for a longer period of time. These experiments are continued until the exact time of clotting is discovered, and this with good technic and experience is quickly arrived at.

The clotting power of the blood is in direct ratio to the time thus discovered. If the clotting power is high, that is, when the time is less than one and a half minutes, and it is deemed necessary to lower it, lemon juice or citric acid is administered. If the clotting power is low, as is shown by the delayed clotting in the tube, calcium lactate is administered with an initial dose of half a dram and 10 grains thrice daily thereafter, articles containing citric acid obviously not being allowed in the diet. In phlebitis, whether thrombosis has occurred or not, citric acid is given in preference to lemon juice and in large doses, one dram as an initial dose followed by 20 grains thrice daily for 4 or 5 days; and after an interval of a few days a resumption of the drug. The reason that the citrates lower the clotting power of the blood is because they combine with the calcium salts, which are essential to the rennet and fibrin ferments. The clotting power is first lessened but, later, by continued use of the citrates the coagulability is again increased, probably owing to the fact that the citrates dissolve the lime salts out of the tissues. In my opinion the estimating of the clotting power of the blood is not only highly scientific but also of great practical benefit. It gives us an indication, if thrombosis has not occurred, not only of the imminence of that troublesome complication, but also of the possible extent to which it may be carried. It further serves the important purpose of indicating exactly when to cease the therapeutic measures which have been instituted, and when to reinstitute them.

It is only in the mildest attacks of phlebitis that thrombosis does not occur; therefore the greatest care should be exercised even in apparently mild attacks. The patient should be put to bed, the limb kept absolutely at rest to limit the inflammation and in an elevated position to assist the venous return. Local remedies, such as belladonna fomentations, may be employed to allay the inflammation and pain. Such precautions and measures should be persevered in until all signs of inflammation have passed away.

*Group 7—Cases which are complicated by the presence of thrombosis.*—As stated above, thrombosis may end in organisation of the clot; pieces of the clot may, however, become detached before organization is completed and swept into the general venous current. When it is consequent on an attack of phlebitis, as it most commonly is, it may break down and suppurate instead of organising.

I will deal with the question of treatment according to the condition present: (*a*) cases which are independent of phlebitis, or are the sequelæ of mild attacks of it; (*b*) cases which are the sequelæ of severe attacks of phlebitis, and pass on into a condition of suppuration.

(a) These cases, taking into consideration the danger of detachment of pieces of the clot, should be treated in a similar manner to that depicted above for the treatment of a simple attack of phlebitis, but those precautions and measures should be prolonged (6 to 8 weeks) until organisation of the thrombus is complete. Massage is then in order to cause absorption of the induration in and around the involved vein. The complete organisation of a thrombus very often affects a cure of the varicosity; in fact it has the same effect that the removal of the veins would have.

(*b*) The greatest caution should be exercised in those cases, in which suppuration is to be feared; it is always wise in such to cut down on to the vein and ligate it well above the site of the thrombosis, so as to insure that pieces of the septic clot will not become detached and swept into the general venous current. Where suppuration has already occurred, operation is demanded; the vein should be exposed by a free incision, ligated well above the site of the suppurating thrombus, and the abscess freely opened. It may be necessary, in the worst cases, to perform extensive operations for the relief of the condition. In those cases seen late a keen watch should be kept up for the appearance of embolic abscesses, which should receive prompt treatment when found.

*Group 8—Cases which are complicated by the occurrence of hemorrhages.*—Hemorrhage, complicating a condition of varix, may be the result of injury, or from an extension of ulceration. Hemorrhage, the cause be it what it may, calls for prompt action; the first aid should consist of pressure firmly and evenly applied; the treatment thereafter varies according to the condition present, but one may take it as a cardinal rule that the occurrence of a hemorrhage is a very strong indication for an operation towards the relief of the varicosity.

327 DELAWARE AVENUE.

---

Mr. McQuire (to hospital attendant)—Phwat did ye say the doctor's name was?

Attendant—Dr. Kilpatrick.

Mr. McQuire—Thot settles it. No doctor wid thot cognomen will git a chance to operate on me—not if I know it.

Attendant—Why not?

Mr. McQuire—Well, ye see, my name is Patrick.—*Judge.*

## Figures and Tables

**Figure f1:**